# Oxiracetam ameliorates cognitive deficits in vascular dementia rats by regulating the expression of neuronal apoptosis/autophagy-related genes associated with the activation of the Akt/mTOR signaling pathway

**DOI:** 10.1590/1414-431X20198371

**Published:** 2019-11-07

**Authors:** Jing Xu, Qianqian Qi, Peiyuan Lv, Yanhong Dong, Xin Jiang, Zhijuan Liu

**Affiliations:** Department of Neurology, Hebei General Hospital, Shijiazhuang City, Hebei Province, China

**Keywords:** Oxiracetam, Vascular dementia, Bilateral common carotid artery occlusion, Autophagy, Apoptosis

## Abstract

Oxiracetam (ORC) is a commonly used nootropic drug for improving cognition and memory impairments. The therapeutic effect and underlying mechanism of ORC in vascular dementia (VaD) treatment remain unknown. In this study, 3-month-old male Sprague-Dawley rats with permanent bilateral common carotid artery occlusion-induced VaD were treated orally with low (100 mg/kg) or high (200 mg/kg) dose ORC once a day for 4 weeks. The results of the Morris water maze test and Nissl staining showed that ORC treatment significantly alleviated learning and memory deficits and neuronal damage in rats with VaD. Mechanistically, the protein levels of a panel of genes associated with neuronal apoptosis (Bcl-2, Bax) and autophagy (microtubule-associated protein 1 chain 3, Beclin1, p62) were significantly altered by ORC treatment compared with VaD, suggesting a protective role of ORC against VaD-induced neuronal apoptosis and autophagy. Moreover, the Akt/mTOR pathway, which is known to be the upstream signaling governing apoptosis and autophagy, was found to be activated in ORC-treated rats, suggesting an involvement of Akt/mTOR activation in ORC-rendered protection in VaD rats. Taken together, this study demonstrated that ORC may alleviate learning and memory impairments and neuronal damage in VaD rats by altering the expression of apoptosis/autophagy-related genes and activation of the Akt/mTOR signaling pathway in neurons.

## Introduction

Vascular dementia (VaD) is the most common cause of dementia in the elderly, second to Alzheimer's disease (AD), and is characterized by progressive cognitive and behavioral deterioration resulting from vascular events that damage blood vessels and impair blood flow in the brain (1,2). Neural lesions and neurodegeneration in various brain regions accompanied by learning and memory deficits have been implicated in VaD (2). Despite recent developments in experimental and clinical neuroscience, no drug has been approved to date for treatment of VaD in the world (3). It is urgently required to find an effective pharmacological compound against VaD due to its high prevalence and morbidity, and lack of effective treatment options.

It is well-established that the balance of protein expression between antiapoptotic Bcl-2 and proapoptotic Bax plays an important role in cell apoptosis (4). A recent study demonstrated that decreased Bcl-2/Bax ratio contributes to neuron apoptosis in the hippocampus of rats with permanent bilateral common carotid artery occlusion (BCCAO), a widely used model of VaD (5,6). In addition, activation of the PI3K/Akt pathway can protect neurons from apoptosis in cerebral ischemia-reperfusion (IR) injury via the regulation of Bax and Bcl-2 expression (7,8). These data suggest Bcl-2/Bax-modulated apoptosis as a potential therapeutic target for prevention and intervention of BCCAO-induced VaD. Furthermore, accumulating evidence indicates that autophagy is another form of programmed neuronal cell death that is triggered by intracellular buildup of pathogenic proteins and damaged organelles (9,10). Modulation of autophagy may serve as an alternative therapeutic strategy in brain disorders such as ischemic stroke, AD, Parkinson's disease, and Huntington's disease (11). Intriguingly, the interplay between apoptosis and autophagy may cause coexistence of both of them in the same cell (12). Mammalian target of rapamycin (mTOR), one of the target genes of the PI3K/Akt pathway, which directly regulates the autophagy cascade, appears to bridge the interaction between apoptosis and autophagy (13,14). Several previous studies have provided some evidence that the activation of Akt/mTOR signaling is involved in the protective effect against vascular cognitive impairment (8,15–18). Various models of IR injury treated with various molecules revealed that IR injury is mitigated through Akt/mTOR signaling (19–24), promoting cell survival.

Oxiracetam (ORC; 4-hydroxy-2-oxo-1-pyrrolidine acetamide), a derivative of γ-aminobutyric acid, is a commonly used nootropic drug for improving cognition and memory impairments (25). ORC can diffuse across the blood-brain barrier and extensively distribute in the hippocampus, cerebral cortex, and striatum with high concentration (26–28), remarkably improving the clinical outcomes of various brain disorders, including neurodegenerative diseases, cognition and memory deficits, seizure, stroke, and anxiety (29). ORC is one of the most common nootropic drugs used for the management of cerebrovascular impairments, including VaD, and has shown beneficial effects (25,30,31). Yao et al. (32) showed that ORC has beneficial effects in rats with cognitive impairment due to chronic cerebral hypoperfusion. Previous studies demonstrated that the beneficial role of ORC in brain disorders is mainly attributable to its abilities to promote the synthesis of lipids, proteins, and nucleic acids as well as increase the levels of high-energy phosphates and membrane-bound protein kinase C in neurons, which leads to an enhancement of metabolism in the brain (33,34). The mechanism of action of ORC is also hypothesized to involve energy metabolism in the brain (33). Nevertheless, whether ORC influences neuronal apoptosis and autophagy is unknown. In addition, whether ORC has a protective role in BCCAO-induced VaD and the underlying mechanisms are poorly understood.

In this study, we established a rat model of BCCAO-induced VaD treated with low- or high-dose ORC to explore the therapeutic effect of ORC on cognition impairments and neuron damage using the Morris water maze (MWM) test and Nissl staining, respectively. To better understand the molecular mechanism underlying ORC-reduced cognitive decline and neuron loss, the expression of a panel of proteins associated with apoptosis and autophagy was determined. The results demonstrated that ORC could improve cognition impairment and brain damage in VaD rats through modulation of apoptosis and autophagy in neuronal cells.

## Material and Methods

### Animals and surgery

Forty Sprague-Dawley male rats (3 months old, 250–300 g) were obtained from the Laboratory Animal Center of Hebei Medical University, China, and maintained in an environmentally controlled room at 23±1°C with a 12-h light/dark cycle and free access to water and food. All animal procedures were approved by the Animal Care and Use Committee of Hebei General Hospital (China) (approval #201903) and carried out in accordance with the Regulations of Laboratory Animal Management issued by the Ministry of Science and Technology of the People's Republic of China.

The rats were randomly divided into four groups (n=10/group): sham group, VaD + saline group (VaD), VaD + low-dose ORC group (ORC-L), and VaD + high-dose ORC group (ORC-H). Rats were anesthetized intraperitoneally (*ip*) with pentobarbital sodium (50 mg/kg), and a 28-gauge single guide cannula was implanted into the left lateral ventricle of each rat using the following coordinates: 0.8 mm posterior to bregma, 1.5 mm lateral to the midline, and 3.8 mm below the skull surface. The rats were allowed to recover for 7 days before BCCAO to induce VaD as previously described (35). Briefly, rats were anesthetized with pentobarbital sodium (50 mg/kg, *ip*). The bilateral common carotid arteries were exposed and gently separated from the carotid sheath and vagus nerve through a ventral cervical incision followed by double-ligation with 4-0 silk sutures and cutting between the ligations. Sham rats underwent the same surgical procedures without occlusion of the carotid arteries. During the surgical procedure, the body temperature was maintained at 36.5–37.5°C using a heat lamp. After surgery, rats were kept in the animal resource facility with free access to food and water.

### ORC administration

ORC powder (99.6% purity) was obtained from Shijiazhuang Pharmaceutical Group NBP Pharmaceutical Co., Ltd. (China) and diluted to 50 mg/mL in physiological saline. One day after BCCAO surgery, the ORC-L and ORC-H groups were orally treated with ORC at 100 and 200 mg/kg, respectively, once a day for 4 weeks, while the sham and saline groups were treated with an equal volume of saline.

### MWM training and test

A circular pool (120 cm in diameter, 60 cm in height; Shanghai Jiliang Software Technology, China), with a video camera hanging over it, was filled to a depth of 45 cm with water at 23±1°C and was located in a dimly lit, quiet test room. Extramaze visual cues were at fixed positions and could be used for spatial orientation by the rats. The maze was divided into four equal quadrants (Northeast, Southeast, Southwest, and Northwest) by placing four poles at the four cardinal points (East, West, North, and South) along the perimeter of the pool. A transparent escape platform was submerged approximately 1 cm below the surface of the water and located in the center of one quadrant during training. From day 1 following drug treatment, each rat received six training trials per day for 5 consecutive days. During each trial, the rats were gently placed in the water and faced toward the wall of the maze from four cardinal points. The time spent to find the hidden platform (escape latency) and the swimming trajectories were recorded. If a rat failed to find the platform within 60 s, the rat would be guided to the hidden platform by the researcher and was allowed to stay on the platform for 15 s before being removed from the water, and a maximum score of 60 s was assigned. Animals with swim speeds less than 10 cm/s were excluded from further analysis due to low motivation and failure to perform the task. On day 6, the hidden platform was removed and the rats underwent a probe test for 60 s. The percentage of time spent in the target quadrant where the platform was located was recorded.

### Nissl staining

At the end of drug treatment, all rats were anesthetized with 1% pentobarbital sodium (50 mg/kg, *ip*) and perfused with 0.9% saline followed by 4% paraformaldehyde through the left cardiac ventricle and ascending aorta. The brain of each rat was immediately removed. Four brain samples were randomly selected from each group, fixed in 4% paraformaldehyde for 48 h, and embedded in paraffin. The hippocampal CA1 area was cut into 5-μm thick sections, and Nissl staining was performed with 1% toluidine blue according to the manufacturer's instructions. Two slides were randomly selected from the same site of each rat and were observed under a 50i light microscope (Nikon, Japan). Images were acquired at ×400 magnification using a Nikon camera (Japan).

### Western blot assay

The hippocampal tissues of six rats randomly selected from each group were removed from the brains and the proteins were extracted using ice-cold radioimmunoprecipitation assay (RIPA) buffer, according to the manufacturer's instructions (Solarbio, China), followed by an incubation on ice for 30 min. The homogenates were centrifuged at 120,000 *g* for 15 min at 4°C, and the supernatants were collected. Protein concentration was measured using the bicinchoninic acid method (Pierce, USA). Protein samples were separated by 12% sodium dodecyl sulfate-polyacrylamide gel electrophoresis and transferred onto polyvinylidene fluoride (PVDF) membranes. After blocking with 5% fat-free milk in Tris-buffered saline and Tween-20 (TBST) for 2 h, the membranes were incubated with primary rabbit antibodies for Akt (1:1000; Epitomics, USA), p-Akt Ser473 (1:1000; Epitomics), Bcl-2 (1:1000; Cell Signaling Technology, USA), Bax (1:1000; Cell Signaling Technology), mTOR (1:500; Epitomics), p-mTOR Ser2448 (1:500; Epitomics), LC3B (1:500; Abgent, USA), p62 (1:2000; Abcam, USA), or β-actin (1:5000, 1:2000; Santa Cruz, USA) overnight at 4°C. β-actin was used as an internal control. Following three washes with TBST, the membranes were incubated with horseradish peroxidase-conjugated secondary antibodies (1:1000, goat anti-rabbit IgG) for 1 h at room temperature. The protein bands on the membranes were detected with the enhanced chemiluminescent reagent (Solarbio). The densitometry values were determined using ImageJ (version 1.3; NIH, Wayne Rasband, USA) and normalized to β-actin.

### Statistical analysis

All data are reported as means±SE. Statistical analysis was performed using SPSS 16.0 (IBM, USA). Differences in the escape latencies in the MWM test were analyzed using the two-way analysis of variance (ANOVA). Statistical significance among multiple groups was assessed using the Student-Newman-Keuls (SNK) test. Other comparisons were conducted using one-way ANOVA followed by the SNK test. P<0.05 was considered statistically significant.

## Results

### ORC ameliorated learning and memory impairments

**Figure 1.**
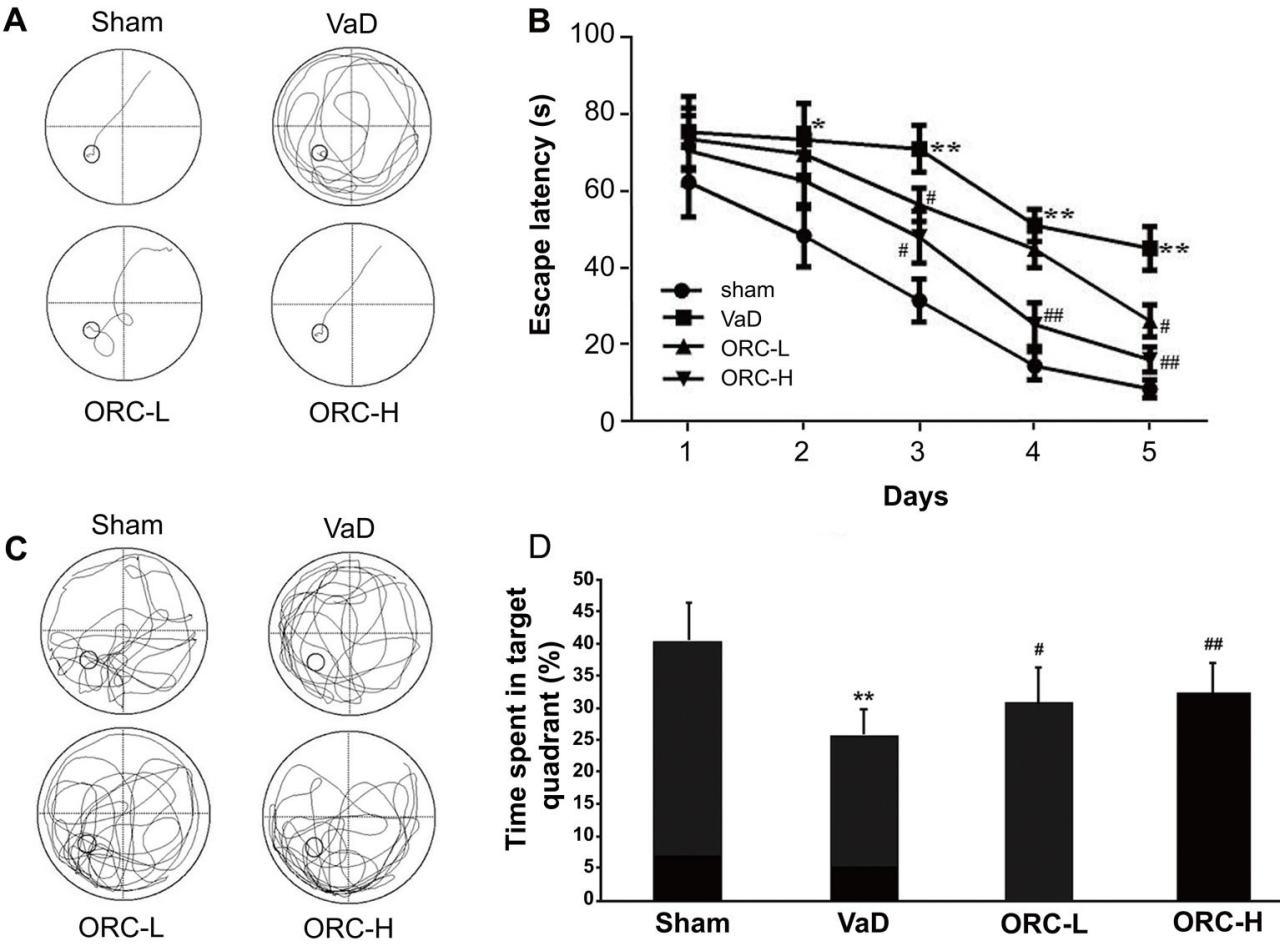
Effects of oxiracetam (ORC) on spatial learning and memory impairments in rats with permanent bilateral common carotid artery occlusion (BCCAO)-induced vascular dementia (VaD) assessed by the Morris water maze test. The swimming trajectories (**A**) and the time spent to find the hidden platform (escape latency; **B**) were recorded daily during training to assess the learning ability. A probe test was performed on day 6 post-ORC treatment to test the memory of the rats. The swimming trajectories (**C**) and the time spent in the target quadrant where the platform was located (**D**) were recorded. *P<0.05, **P<0.01 *vs* sham group; ^#^P<0.05, ^##^P<0.01 *vs* saline (VaD) group; n=10 for each group (ANOVA). ORC-L: low-dose oxiracetam; ORC-H: high-dose oxiracetam.

The results of the MWM test showed that the swimming trajectory length and escape latency in the VaD group were significantly increased compared with those in the sham group, which indicated learning impairment in rats with BCCAO-induced VaD. Compared with the VaD group, ORC treatment significantly decreased swimming trajectory length and escape latency as early as 3 days post treatment in both ORC-treated groups in a dose-dependent manner (F=51.132 for intergroup comparison; P<0.05) (Figure 1A and B), suggesting that ORC could improve the learning ability of rats with VaD. Furthermore, in the probe test, rats in the VaD group spent significantly less time in the target quadrant than those in the sham group (F=15.009; P<0.01), and rats in the ORC-L and ORC-H groups spent more time than those in the VaD group (F=15.009; P<0.01) (Figure 1C and D), suggesting a memory-improving effect of ORC in VaD rats. Taken together, these data demonstrated that ORC may be an effective therapeutic agent in reducing learning and memory deficits in rats with VaD.

### ORC reversed neuropathological alterations

**Figure 2.**
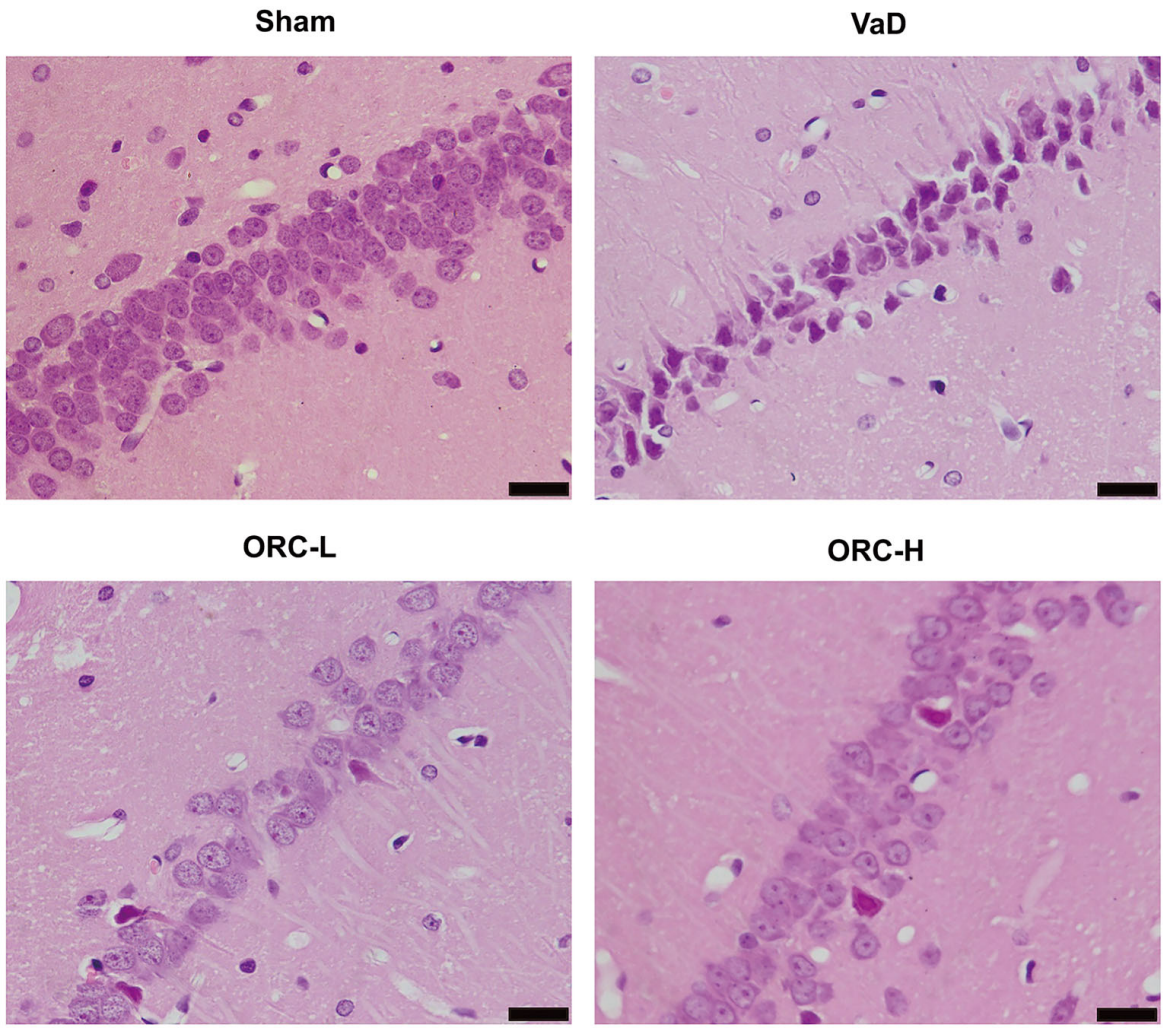
Nissl staining in the hippocampus CA1 region of rats. Magnification 400×; scale bar: 20 μm. ORC-L: low-dose oxiracetam; ORC-H: high-dose oxiracetam; VaD: vascular dementia. In the sham group, neuron cells with normal size and morphology, clear nucleus, and Nissl substance in the cytoplasm were tightly packed and orderly arranged. In the VaD group, obvious pathologic changes were evident, including loosely arranged neurons, neuronal shrinkage and loss. These neuropathological changes and neuron loss were significantly improved in ORC-treated mice, and the improvement in the ORC-H group was more obvious than ORC-L group.

In the sham group, neuron cells with normal size and morphology, clear nucleus, and Nissl substance in the cytoplasm were tightly packed and orderly arranged. In contrast, neurons with loss of Nissl substance and abnormal nuclear shape were loosely and irregularly arranged in the VaD group. In addition, there was a significant loss of neurons observed in the saline group compared with the sham group (Figure 2). These neuropathological changes and neuron loss were significantly improved in ORC-treated mice, suggesting a protective role of ORC against neuronal damage in VaD.

### ORC altered the expression of apoptosis/autophagy-related genes in the hippocampus of rats

**Figure 3.**
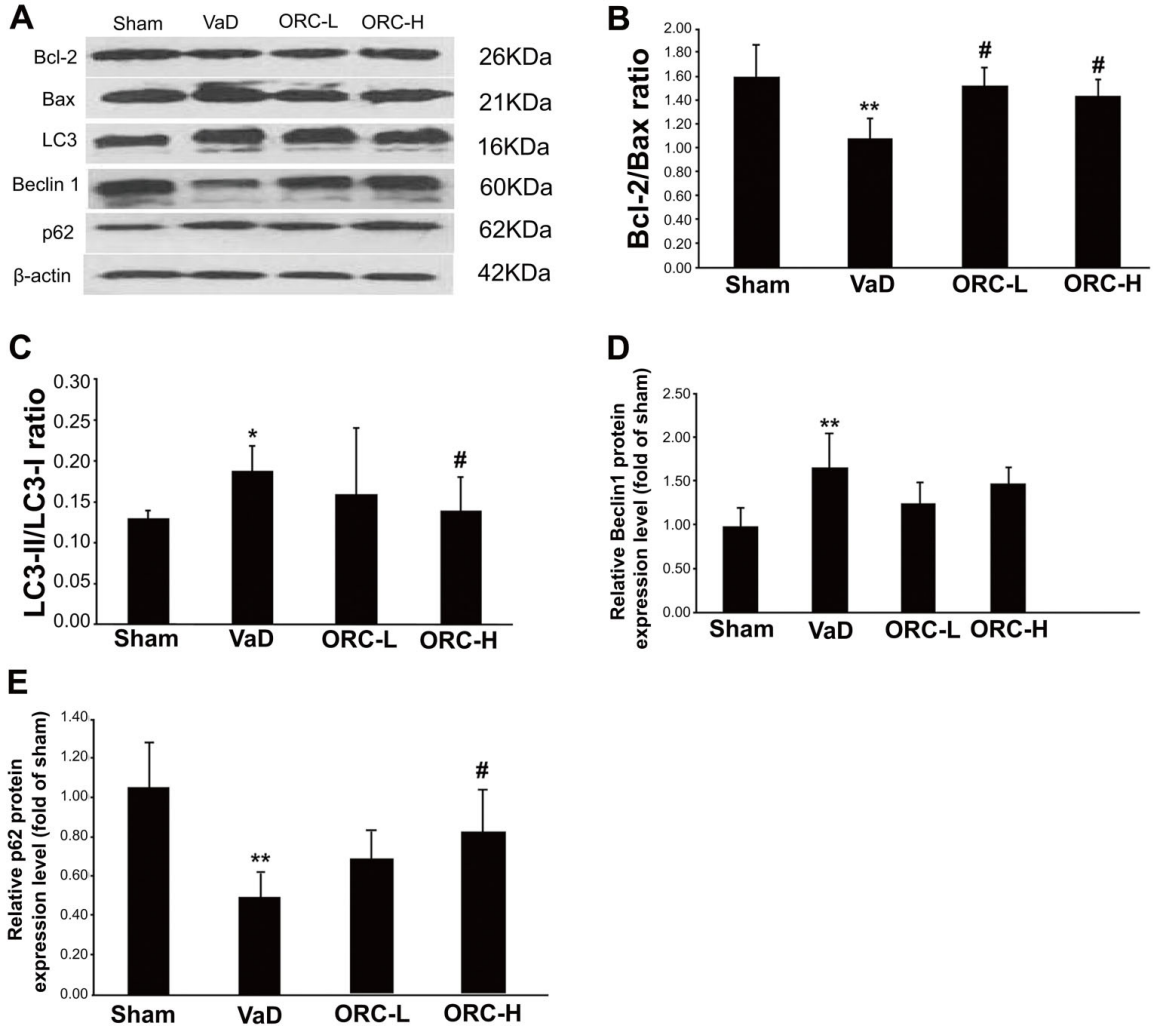
Alteration of apoptosis- and autophagy-related gene expression in response to oxiracetam (ORC). **A**, The protein levels of Bcl-2, Bax, LC3, Beclin1, and p62 in the hippocampus of rats were determined by western blot analysis after 4 weeks of ORC treatment. **B**, Bcl-2/Bax ratio. **C**, LC3-II/LC3-I ratio. **D** and **E**, Quantification of Beclin1 and p62 protein expression. β-actin was used as the internal control. *P<0.05, **P<0.01 *vs* sham group; ^#^P<0.05, ^##^P<0.01 *vs* VaD group; n=10 for each group (ANOVA). VaD: vascular dementia; ORC-L: low-dose oxiracetam; ORC-H: high-dose oxiracetam; LC3: microtubule-associated protein 1 chain 3; LC3-II: phosphatidylethanolamine-conjugated LC3.

As shown in Figure 3A and B, the decreased Bcl-2/Bax ratio in the VaD group was significantly restored by ORC treatment regardless of dose, suggesting an inhibitory role of ORC in neuronal apoptosis. In addition, the conversion of microtubule-associated protein 1 chain 3 (LC3) to phosphatidylethanolamine-conjugated LC3 (LC3-II), which reflects the number of autophagosomes or the degree of autophagy (36), was significantly decreased in the ORC-H group (F=15.577; P<0.05), but not in the ORC-L group, compared with that in the VaD group (Figure 3C), suggesting a protective role of ORC against autophagy at the high dose. Similar trends were also observed in the expression of other autophagy-related genes, such as Beclin1 and p62 (Figure 3D and E). Taken together, these data indicated that ORC may protect neuron cells from apoptotic and autophagic cell death through altering the expression of a variety of related genes.

### ORC induced phosphorylation of Akt and mTOR in the hippocampus of rats

**Figure 4.**
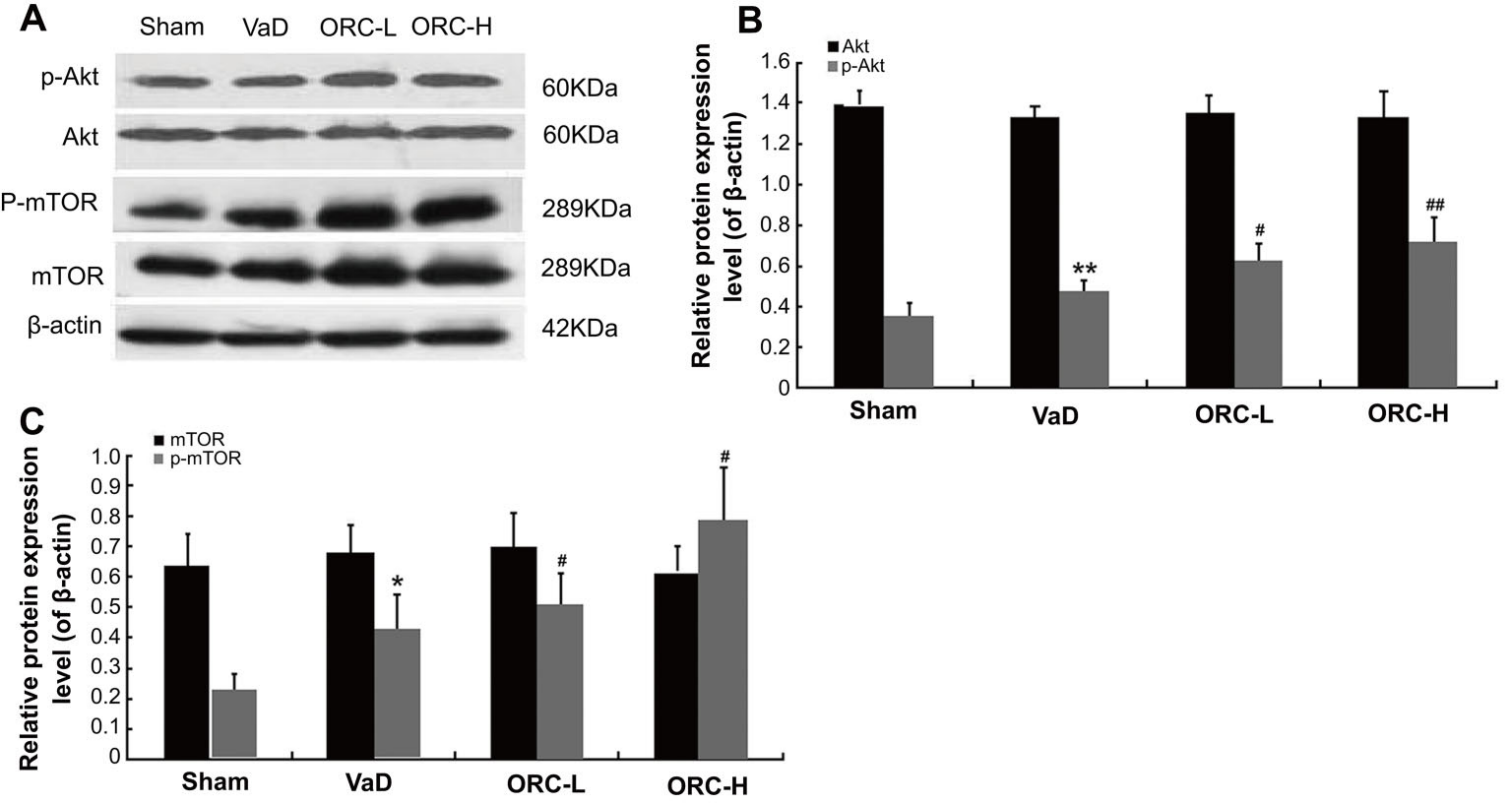
Alteration of apoptosis- and autophagy-related gene expression in response to oxiracetam (ORC). **A**, The protein levels of total Akt, phosphorylated Akt (p-Akt), total mTOR and phosphorylated mTOR (p-mTOR) in the hippocampus of rats were determined by western blot analysis after 4 weeks of ORC treatment. **B** and **C**, Quantification of panel **A**. β-actin was used as an internal control. *P<0.05, **P<0.01 *vs* sham group; ^#^P<0.05, ^##^P<0.01 *vs* saline (VaD) group; n=10 for each group (ANOVA). VaD: vascular dementia; ORC-L: low-dose ORC; ORC-H: high-dose ORC; mTOR: mammalian target of rapamycin.

Considering that Akt signaling is involved in neuronal apoptosis and that mTOR bridges the interplay between apoptosis and autophagy (13,14), we next sought to investigate whether ORC could change the levels of activated Akt and mTOR to reveal the signaling upstream ORC-suppressed apoptosis/autophagy. As shown in Figure 4 A–C, both phosphorylated Akt (p-Akt) and phosphorylated mTOR (p-mTOR) were significantly increased in ORC-treated groups (p-Akt: F=25.301; p-mTOR: F=21.183; both P<0.05) compared with the VaD group in a dose-dependent manner, while the protein levels of total Akt and total mTOR remained unchanged. These data suggested that the protective role of ORC against BCCAO-induced neuronal apoptosis/autophagy was mediated by the activation of the Akt/mTOR signaling pathway.

## Discussion

VaD is characterized by progressive cognitive impairment and extensive neuropathological lesions in brain tissues due to chronic cerebral hypoperfusion (1–3,37). An effective therapeutic approach is urgently required for reducing cognitive degeneration and neuronal damages in VaD. In this study, the results of the MWM test showed remarkable poorer learning and memory skills of rats in the saline group than those in the sham group. A consecutive daily administration of ORC at 100 or 200 mg/kg dose-dependently improved the impaired learning and memory abilities of VaD rats as evidenced by markedly decreased escape latencies in the training sessions and increased time spent in the target quadrant in the probe trial compared with the saline group. Similar trends were also observed in Nissl staining for neurons. These results are consistent with previous research (38–40), suggesting that the rat model of VaD used in the present study is reliable.

To explain why ORC treatment can reduce neuron damage and loss in the hippocampus of VaD rats, as shown by Nissl staining, we explored the expression of a variety of genes associated with apoptosis and autophagy, as they are two major processes involved in neuronal damages and death (5,6,9,10). Several previous studies have provided some evidence that the activation of Akt/mTOR signaling is involved in the protective effect against vascular cognitive impairment (8,15–18). Compared with the sham group, the VaD groups showed some baseline activation of Akt/mTOR signaling, which is probably a response to the IR injuries. Consistent with previous studies showing that the imbalance between antiapoptotic Bcl-2 and apoptotic Bax in repeated cerebral IR and chronic cerebral hypoperfusion are two common events in VaD animal model (5,41,42), we found that the ratio of Bcl-2/Bax was significantly decreased in VaD rats compared with the sham ones, suggesting an enhancement of neuronal apoptosis in VaD rats. Interestingly, the decreased ratio of Bcl-2/Bax in VaD rats was significantly restored upon treatment with ORC regardless of dose, suggesting a neuroprotective role of ORC against apoptotic cell death. The present study suggests that oxiracetam treatment could further increase AKT/mTOR signaling to protect the cells against apoptosis and autophagy, as shown in various models of IR injury and various molecules used to mitigate its damage (19–24), and hence promoting cell survival. Moreover, dysregulation of autophagy markers, including LC3, Beclin1, and p62, was observed in the hippocampus of VaD rats compared with sham ones, suggesting an involvement of autophagy in VaD-induced neuron damage and loss. Although autophagy is primarily a protective process for cell survival, it seems that autophagy has a dual role in the central nervous system (43). Excessive autophagy exerts a detrimental effect in neurological disease, leading to autophagic neuronal death (10). ORC-induced reversal of dysregulated expression of LC3, Beclin1, and p62 indicates that targeting autophagy is another possible mechanism underlying the protective role of ORC in VaD. However, the exact role of autophagy in VaD needs further investigation.

To further clarify the signaling pathway upstream ORC-regulated apoptosis/autophagy, the phosphorylation status of Akt/mTOR was assessed because Akt/mTOR signaling is involved in the interplay between apoptosis and autophagy (13,14). It has been reported that phosphorylation of Akt can suppress IR-induced apoptotic cell death in neurons through upregulation of antiapoptotic genes and downregulation of proapoptotic ones (44). Our results showed that ORC treatment significantly upregulated p-Akt and p-mTOR expression in VaD rats, suggesting an involvement of Akt/mTOR activation in ORC-rendered protection against VaD-induced apoptosis/autophagy.

In conclusion, the present work demonstrated that ORC could attenuate cognitive impairment and neuronal damages in BCCAO-induced VaD in rats possibly through regulation of neuronal apoptosis- and autophagy-related genes via activation of the Akt/mTOR signaling pathway, which provides ORC as an effective therapeutic compound targeting neuronal apoptosis/autophagy in VaD treatment. Further studies are required to reveal the precise effect and mechanism of ORC in VaD therapy.
